# Health management information system (HMIS) data quality and associated factors in Massaguet district, Chad

**DOI:** 10.1186/s12911-021-01684-7

**Published:** 2021-11-22

**Authors:** Azoukalné Moukénet, Monica Anna de Cola, Charlotte Ward, Honoré Beakgoubé, Kevin Baker, Laura Donovan, Jean Laoukolé, Sol Richardson

**Affiliations:** 1 Malaria Consortium Chad Country Office, Angle Bureau de L’Entente Des Eglises (EEMET), Rue 2175, Porte 0150, B.P. 6180 N’Djamena, Chad; 2 Malaria Consortium, The Green House, 244-254 Cambridge Heath Road, London, E2 9DA UK; 3 Department of Global Health and Development, Faculty of Public Health and Policy, London School of Hygiene and Tropical Medicine, London, UK; 4 National Malaria Control Programme, N’Djamena, Chad

**Keywords:** Data quality, Health management information system, Malaria, Chad

## Abstract

**Background:**

Quality data from Health Management Information Systems (HMIS) are important for tracking the effectiveness of malaria control interventions. However, HMIS data in many resource-limited settings do not currently meet standards set by the World Health Organization (WHO). We aimed to assess HMIS data quality and associated factors in Chad.

**Methods:**

A cross-sectional study was conducted in 14 health facilities in Massaguet district. Data on children under 15 years were obtained from the HMIS and from the external patient register covering the period January–December 2018. An additional questionnaire was administered to 16 health centre managers to collect data on contextual variables. Patient registry data were aggregated and compared with the HMIS database at district and health centre level. Completeness and accuracy indicators were calculated as per WHO guidelines. Multivariate logistic regressions were performed on the Verification Factor for attendance, suspected and confirmed malaria cases for three age groups (1 to < 12 months, 1 to < 5 years and 5 to < 15 years) to identify associations between health centre characteristics and data accuracy.

**Results:**

Health centres achieved a high level of data completeness in HMIS. Malaria data were over-reported in HMIS for children aged under 15 years. There was an association between workload and higher odds of inaccuracy in reporting of attendance among children aged 1 to < 5 years (Odds ratio [OR]: 10.57, 95% CI 2.32–48.19) and 5– < 15 years (OR: 6.64, 95% CI 1.38–32.04). Similar association was found between workload and stock-outs in register books, and inaccuracy in reporting of malaria confirmed cases. Meanwhile, we found that presence of a health technician, and of dedicated staff for data management, were associated with lower inaccuracy in reporting of clinic attendance in children aged under five years.

**Conclusion:**

Data completeness was high while the accuracy was low. Factors associated with data inaccuracy included high workload and the unavailability of required data collection tools. The results suggest that improvement in working conditions for clinic personnel may improve HMIS data quality. Upgrading from paper-based forms to a web-based HMIS may provide a solution for improving data accuracy and its utility for future evaluations of health interventions. Results from this study can inform the Ministry of Health and it partners on the precautions to be taken in the use of HMIS data and inform initiatives for improving its quality.

**Supplementary Information:**

The online version contains supplementary material available at 10.1186/s12911-021-01684-7.

## Background

The World Health Organization (WHO) has highlighted the importance of investing in high-quality routine information systems to support malaria surveillance and monitoring of malaria program activities [1], and complement overall malaria elimination efforts [2]. In addition, the WHO emphasizes the importance of quality of data across nine dimensions: accuracy, validity, reliability, completeness, legibility, timeliness, accessibility, usefulness and confidentiality [3]. However, Health Management Information Systems (HMIS) in many resource-limited settings, including Chad, do not currently meet these standards [4, 5]. Hence, sub-Saharan Africa countries where malaria is endemic are placing greater priority on the quality and utility of the malaria data generated by their HMIS [6–8]. Trends in malaria incidence can be used to evaluate impact of malaria interventions such as Seasonal Malaria Chemoprevention (SMC) and support decision-making on their introduction and expansion. While HMIS is a convenient and readily-available source for such data, it is important to ensure the HMIS data is sufficiently reliable decision-making.

In Chad, HMIS and other malaria data for Malaria Programme are routinely collected by health facilities from communities in their catchment area and from routine consultations and transactions using paper forms, which are then verified by health facility managers before being reported to the health district. At the district level, data from health facilities and district hospitals are converted to electronic format (Microsoft Excel) by the zonal chief or district manager. From the district, data are sent by email or flash key to the Provincial Health Delegation, and finally to the Department of Statistics and Health Information (SDSIS) of the Ministry of Health. In addition, malaria data are sent to the National Malaria Control Program (NMCP). At the central level, the SDSIS verifies the consistency of HMIS data.

In contrast to HMIS data, at the central level, the NMCP, in collaboration with district managers, province data managers and partners in malaria control, validates data during quarterly meetings in addition to monthly supervision visits at selected health facilities and health districts. To ensure the quality of data collected and the malaria case management, the NMCP, SDSIS and Provincial Health Delegation conduct supportive supervisions at districts and health centres. Although health facility personnel are expected to validate data before submission for onward collation, and as the health facilities and health districts receive monthly supervision visits, there is potential for transcription errors.

Many factors are associated with data quality issues, including availability of reporting tools; most HMIS in sub-Saharan Africa are paper-based at the health facility level involving manual collection and collation of data. Evidence suggests that the continued use of paper-based systems contributes to poor data quality in terms of reliability, availability, timeliness and completeness of reporting, and can compromise health service delivery [9]. Other authors have highlighted that the primary point of departure for accurate data transfer is during the tallying and collation process at the facility level, between the registers and routine monthly reports [10]. At that level, there are multiple registers and tally sheets that need to be collated, summarized and sent to the next level [11]. Other technical reasons for poor data quality include use of inappropriate data collection tools and procedures that result in recording and reporting practices which undermine standardization [12–14]. For example, difficulties in differentiating between suspected and confirmed malaria cases may contribute to misreporting of malaria cases [15, 16]. In addition, stock-outs of paper forms are a recurrent problem in Chad and across sub-Saharan Africa [7, 8, 17, 18], leading to the development of improvised tools which sustain the data collection process but have implications for data quality.

In addition to technical factors related to lower data quality, there are human resource factors; supervisors often have very limited data quality checking skills, may not understand the importance of collecting data, and may not provide feedback after supervision [10, 19]. In Chad, as in other sub-Saharan African countries, there are shortages of healthcare workers that compromise the quality of data collected [12, 17, 19]. In addition, the occurrence of incidents such as epidemic disease outbreaks can increase the workload for health staff and lead to collection of lower quality data. Also, others incidents such as strikes and flooding can divert attention of data managers during data validation since health staff are focussed on managing incidents. Often health workers in the field have inadequate skills for routine data collection, and may not have received relevant training [13]. In addition, they are poorly paid and often experience delays in salary payments, which affect their morale [12] and lead to staff attrition [10, 13]. In some countries, as part of performance evaluations, governments emphasize the need to improve maternal and child health and reduce malaria incidence, and staff may be incentivized to over-report delivery of maternal and child health services and underreport malaria cases [19].

Although some authors advise caution when using HMIS data [6], it is recognized that HMIS data are readily accessible and a low-cost solution for assessing the impact of health programmes, particularly since randomized approaches may not be appropriate or ethical [20]. In Chad, SMC has been implemented to prevent malaria among children aged 3–59 months since 2015 with support from partners including Malaria Consortium who provide operational support to SMC delivery and ensure supervision, monitoring and evaluation. While SMC’s efficacy is well recognized, HMIS is considered a useful data source for assessing the impact of this intervention when implemented at scale. Thus, the low quality of HMIS data may be a source of bias for impact evaluations and lead to decisions based on wrong evidence in context where resources for health become scare. In addition, Chad is assessing the feasibility of expanding SMC at scale, both to new areas of the country and to school age children as in other similar settings [21, 22]. HMIS is a ready available and valuable source of data on trends on malaria incidence which can be employed for decision-making for SMC’s expansion [23]. Therefore, researchers and program managers would be interested in knowing the current quality of HMIS data given their utility for decision-making and the evaluation of the impact of SMC and other malaria control programs.

Supervision reports have identified quality issues in the Chadian HMIS [17, 24]. To our knowledge, no study has been conducted in Chad to assess HMIS data quality and the factors associated with data quality. To inform the Ministry of Health and its partners on the precautions to taken in the use of HMIS data and identify main areas for improvement, this study aimed to assess current gaps in the quality of data submitted for selected malaria indicators in HMIS and identify factors influencing data accuracy.

## Methods

### Study setting

The study was conducted in the health district of Massaguet in Hadjer Lamis Province, which includes 16 health centres and has been implementing SMC for children aged 3–59 months since 2015 with support from Malaria Consortium. In 2017, a study found that malaria prevalence in Massaguet was 15.9% which is higher than the national average for SMC eligible districts (7.7%) [25]. The study was realized alongside with another aimed to assess the feasibility of SMC extension to older children (> 5 years) in Chad. Massaguet district was chosen for operational reasons (proximity to N’Djamena) and convenience with the National Malaria Control Program (NMCP).

As part of malaria case management, health centres in Chad are supplied with antimalarial medicines and rapid diagnostic tests (RDT) by the NMCP, under the *programme d’appui à la lutte antipaludique au Tchad* (PALAT), with financial support from the Global Fund [26]. NMCP is responsible for supplying health centres with tools for data collection such as malaria data collection forms and monthly malaria report forms, in addition to short training (2–5 days) for health centre staff on data management and malaria case management. In addition, all training curricula for clinicians in Chad include malaria case management and data management for malaria. To support malaria data management activities, health centre staff receive a bonus each month. In practice, many health centres experience stock-outs of data collection tools and commodities, and delays in bonus payments.

### Participants

A total of 16 health centre managers in Massaguet (one per health facility) were surveyed to obtain data on contextual factors relating to their health facilities. All types of health centre in Massaguet were represented (public, community and confessional).

### Data collection and sources

The data collection consisted of three parts:

#### Recounted data from paper based registers from health facility

Routine clinical data from paper-based registers covering January–December 2018 were extracted from the external consultation patient register in April 2020 (30 days) at 14 government-recognized health centres in Massaguet district. Data were entered by four trained researchers into an electronic questionnaire form (which can be administered offline with responses uploaded to the server once WiFi connection is available) (see Additional file 2) using Magpi v6.2.1 software [27]. The questionnaire consisted of two forms. While the first was used to collect data on the population of the health facility’s catchment area and stock-outs of RDTs for each month of the study period, the second included four sections with questions relating to each patient aged under 15 years old who attended the health facility covering their socio-demographic characteristic, place of residence, clinical health problems identified, treatment administered/advice prescribed, and outcomes (i.e. referral or death). Thus, extracted data from this source included variables on suspected and confirmed malaria cases (by RDT and microscopy) among children under 15 years old. Demographic variables of malaria cases included age, sex, and place of residence (≤ five kilometres (km) from the health facility, > 5 km from health facility, nomadic, within catchment area of another health centre). In addition, data on treatment prescribed and clinical outcomes (death and referral to hospital) were extracted. To preserve anonymity, an ID code was given to each patient on which clinical data were extracted.

#### HMIS data

The HMIS data for January–December 2018 at health centre level and district-level were obtained from the NMCP.

#### Survey data on contextual factors

In addition, an electronic questionnaire (see Additional file 3) was administered to all health centre managers in Massaguet district using Magpi v6.2.1 to collect data on contextual variables relating to the health facility. The questionnaire included items on the health facility and its location, the availability of facilities and amenities (electricity, water, phone, system for waste, type of rooms, etc.), the situation of staff (type of staff available, perception of workload, training provided to staff, etc.), occurrence of incidents with the potential to disrupt services, and the availability of commodities. Thus, for the purposez of this study variables retained include health centre type (public, community and confessional), location (urban, rural and peri-urban), data on human resources including number and type of staff employed (nurse, mid-wife, health agent, and health technician), perceptions of levels of staffing on health centre performance, and availability and training of staff dedicated to malaria data management. For each month, data on arrears in bonuses for malaria data reporting and data on working conditions, including occurrence of incidents (strikes, flooding, community violence, epidemic disease outbreak) and stock-outs of commodities (RDTs, antimalarial drugs, register books, HMIS forms, Monthly Malaria Report (RMP) forms), were recorded. The questionnaire form is included as an online supplement (Additional file 3). Researchers were trained on data collection and all tools were piloted in the health centre of Abena in N’Djamena Sud district before use in the field. The consultation register of that centre was used to test the data audit.

### Data analysis

#### Variable definitions

Extracted patient registry data were aggregated and compared with the HMIS database. Data from the patient registry data and HMIS were assessed on the dimensions of completeness and accuracy respectively at two levels: (i) the district-level, between aggregated data extracted from health centre patient registry to the HMIS data validated at the NMCP, and (ii) the health centre level, between aggregated data extracted from health centre patient registry data to the HMIS data compiled at the health centre level.

#### Completeness of indicator data (data element)

This is defined as “the extent to which facilities that are supposed to report data on the selected core indicators are doing so” [28]. In this study, completeness of reporting was calculated by adding the number of months with values reported for a given indicator across all health facilities in a year divided by the expected number of values (i.e., 12 months × total number of health facilities). Any missing fields for a specific outcome and month were considered to be unreported data.

#### Consistency of reported data and original records, or data accuracy

Data accuracy was assessed as per the WHO data quality review guidelines [28] through verification factors (VFs) for each indicator. A VF was defined as the ratio of an indicator in registry data divided by that of HMIS data at the health centre level. We calculated VFs at the health centre level for all patient attendances, suspected malaria cases, and confirmed malaria cases. These indicators were disaggregated into the following age groups: 1 to < 12 months, 1 to < 5 years, and 5 to < 15 years. For each indicator (attendance, suspected and confirmed malaria cases), we excluded any health centre not yet recognized by the government and health centres for which a given indicator was completely missing in registry data during the reporting period.

A VF value of 1 represents a perfect match between data from the patient registry and HMIS. The acceptable margin of error for the discrepancy between HMIS report data and recounted data from health centre registers was defined as a VF value within 0.90–1.10 as per the WHO Data Quality Review guidelines. VFs of < 0.90 or VF > 1.10 indicated under-reporting and over-reporting of cases in HMIS data respectively [28]. For each month and each indicator (attendance, and suspected and confirmed simple malaria cases), health centres were classified into two categories according to their VF value (i.e. within the interval or outside the 0.90–1.10 interval).

### Statistical analysis

We conducted univariate descriptive analyses to present characteristics of surveyed health centres, and completeness and accuracy of outcome indicators.

We fitted multivariate logistic regression models to identify associations between health centre characteristics and data accuracy; odds ratios (OR) and 95% confidence intervals (95% CI) were calculated. In the first step of the regression, we purposively included all the variables we hypothesized were associated with data accuracy. These included variables related to human resources such as skills [13] and workload [12, 17, 19], and design, structure [12–14] and availability of tools [7, 17, 18, 29]. To assess skills, we fitted “presence of nurse and health technician within staff”, “presence of a staff member dedicated to data management”, and “presence of staff trained in data management”. For workload, we fitted a binary variable corresponding to the months of the rainy season (July–October), the period when health facilities are implementing SMC and when the attendance is high, resulting in high workload. We also fitted variables for months of occurrence of stock-outs of register books, malaria data collection forms and HMIS forms. Finally, in 2018, there were changes to malaria data collection forms with new requirements for data reporting (this was operationalized as a binary variable).

The second step of regression added additional variables using a forward stepwise procedure in which further covariates were added based on improvement of model fit as determined using the likelihood ratio test. These variables included availability of commodities and medicines such as antimalarial drugs, RDTs, rooms for private consultation, arrears in payment of incentives for data collection, and others (see Additional file 1).

Regression analyses were performed separately for VFs for three indicators (attendance, suspected malaria cases and confirmed simple malaria cases) for each of the three age groups (1 to < 12 months, 1 to < 5 years and 5 to < 15 years). All data management and analyses were performed using Stata 16.

## Results

The descriptive analysis included data from all 16 health centres of Massaguet (14 registered and 2 non-registered health centres). The statistical analysis covered 14 health centres in Massaguet, with monthly observations covering the period January–December 2018, excluding non-registered health centres. Furthermore, one health centre missed two months of patient registers. The final dataset therefore covered 12 health centres for 12 months and one health centre (Karme health centre) for 10 months, resulting in a total of 154 monthly observations.

### Characteristics of health centres surveyed

There was an average of 4.94 (95% CI 3.91–6.15) staff per health centre, including non-clinicians. Health centre managers reported 6.25% of facilities to be “severely understaffed”, and 43.75% of facilities “somewhat understaffed” (Table 1). Unavailability of commodities influenced functioning of health centres; 56.25% of health centres sampled reported at least one stock-out of RDT, with an average of 16.53 days duration (CI 14.54–18.72) across all facilities over the whole study period. Concerning tools for data collection, over the study period, there were stock-outs of register books at 37.50% of health centres (mean: 30.32 days, CI 27.89–32.90), HMIS forms at 12.50% of health centres (mean: 26.83 days, CI 22.85–31.31), and malaria data collection forms at 18.75% of health centres (mean: 23.13 days, CI 19.91–26.71). Half of the health centres surveyed (50.00%) had a person dedicated to data management. See Additional file 1 for additional descriptive data on sampled health facilities.

**Table 1:** Characteristics of health centres surveyed

Variables		Freq	Percent	Mean (95% CI)
Presence of nurse within staff (N = 16)	Yes	7	43.75	
Presence of midwife within staff (N = 16)	Yes	7	43.75	
Presence of technical agent within staff (N = 16)	Yes	15	93.75	
Presence of technical health agent within staff (N = 16)	Yes	10	62.50	
Stock-out of RDT(N = 16)	Yes	9	56.25	
Stock-out of antimalarials (N = 16)	Yes	9	56.25	
Stock-out of registers (N = 16)	Yes	6	37.50	
Stock-out of HMIS forms (N = 16)	Yes	2	12.50	
Stock-out of malaria data collection form (N = 16)	Yes	3	18.75	
Staff dedicated to data management (N = 16)	Yes	8	50.00	
Staff dedicated for data trained (N = 16)	Yes	9	56.25	
New data collection form and reporting requirements, issued 2018 (N = 16)	Yes	4	25.00	
Staff trained in RDT management (N = 16)	Yes	12	75.00	
Number days of stock-outs of RDT (N = 192)				16.53
				(14.54–18.72)
Number days of stock-outs of antimalarials (N = 192)				21.13
				(18.93–23.50)
Number days of stock-outs of registers (N = 192)				30.32
				(27.89–32.90)
Number days of stock-outs of HMIS forms (N = 192)				26.83
				(22.85–31.31)
Number days of stock-outs of malaria data collection forms (N = 192)				23.13
				(19.91–26.71)
Training duration (N = 16)				2.75
				(1.72–4.16)

### Completeness and accuracy of data reporting in HMIS

Completeness of reporting of malaria indicators (attendance, suspected malaria and confirmed simple malaria) for considered age group was 95.24% (CI 90.83–97.92) for the HMIS data and 98.81% (CI 95.80–99.90) for patient registry data. There were no missing values for specific indicator; in case of missing values it was missing completely for all indicators.

However, during the study period there was an over-reporting in attendance, and suspected and confirmed malaria cases in HMIS for children aged under 15 years with VF values between 1.41 and 2.86 (Table 2). Over-reporting was higher for suspected malaria cases than other indicators. A comparison of VF per age group and indicator shows higher over-reporting of indicators for older children.

**Table 2:** Verification factors for attendance, suspected and confirm simple malaria cases by age group at health centre level (N = 192)

	0 to < 12 m	1 to < 5 y	5 to < 15 y	All
Attendance	1.21	1.36	1.64	1.41
Suspected malaria	2.48	2.70	3.41	2.86
Confirmed simple malaria	1.10	2.16	2.55	1.94

**Fig. 1:**
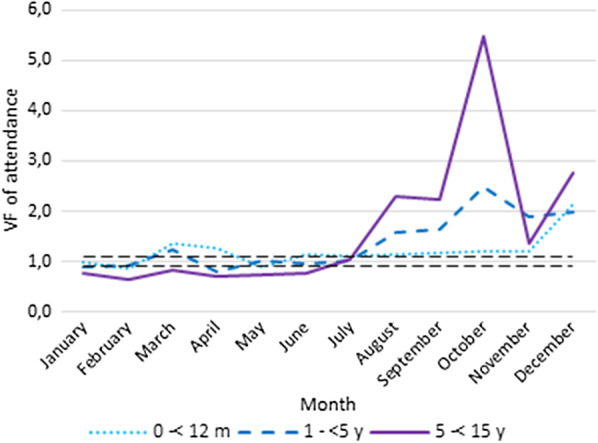
Verification factors of attendance by age group and per month

**Fig. 2:**
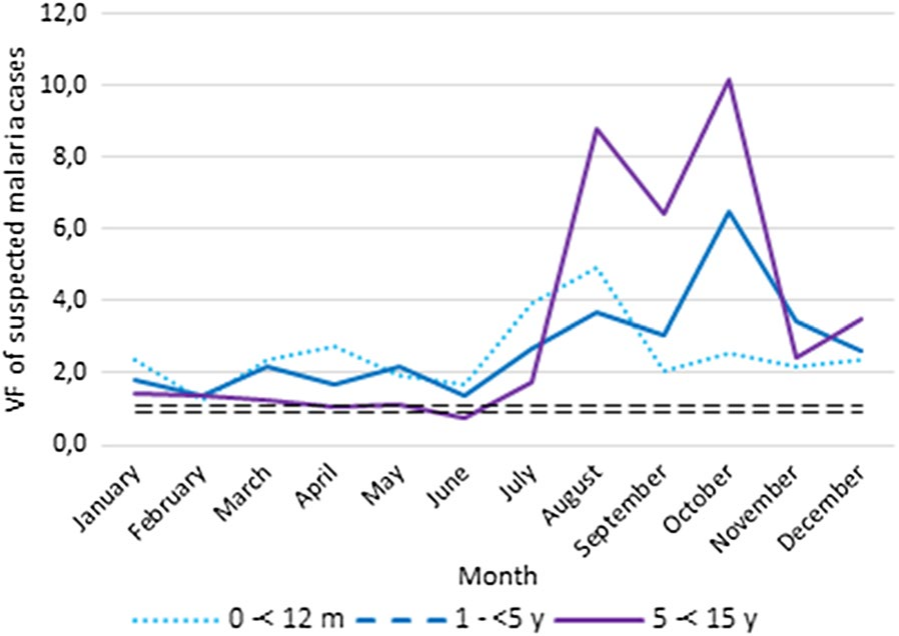
Verification factors of suspected malaria cases by age group and per month

**Fig. 3:**
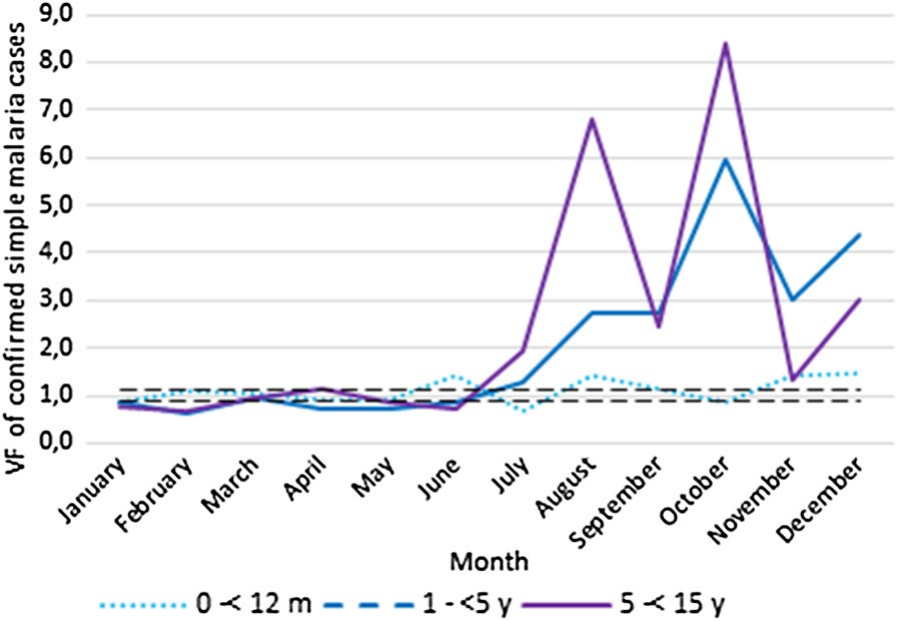
Verification factors of confirmed simple malaria cases by age group and per month

In general, all health centres over-reported all indicators in HMIS. VF values varied by health centre and indicator. In 2018, the degree of data inaccuracy reported was higher during the raining season (July–October) (Figs. 1, 2, 3).

### Factors associated with inaccuracy in HMIS data reporting

Accuracy of attendance reporting data is associated with the presence of skilled staff, the introduction of new malaria data reporting requirements, staff workload, and stock-outs of tools and malaria RDTs. Respectively, presence of a health technician and staff dedicated to data management were associated with significantly lower odds of inaccurate reporting of attendance among children aged 0 to < 12 months in HMIS (OR: 0.09, 95% CI 0.01–0.86 and OR: 0.01, 95% CI 0.00–0.30 respectively); associations were borderline-significant for children aged 1 to < 5 years (OR: 0.14, 95% CI 0.01–1.36 and OR: 0.06, 95% CI 0.00–1.12 respectively). Stock-outs of HMIS forms were associated with lower odds of inaccurate reporting of data on under five years age’s attendance into HMIS; OR: 0.03 (95% CI 0.00–0.73) for children 0 to < 12 months and OR: 0.03 (95% CI 0.00–0.44) for 1 to < 5 years. There was a significantly higher odds of inaccurate reporting data on attendance of children aged 1 to < 5 years during the months of the rainy season (10.57, 95% CI 2.32–48.19; 6.64, 95% CI 1.38–32.04). The changes to the malaria data collection form and new data reporting requirements were associated with the higher odds (5.66, 95% CI 0.82–39.20; 24.28, 95% CI 0.75–791.45) of inaccurate reporting of attendance data for children aged 0 to < 12 months and 5 to < 15 years (Table 3).

**Table 3:** Multivariate analysis for associations of clinic-level variables with inaccurate reporting of data from register into HMIS (N = 154), (odds ratios shown with 95% confidence intervals in parenthesis)

Variables	Attendance at a health centre			Suspected malaria cases			Confirmed malaria cases		
	0 to < 12 m	1 to < 5 y	5 to < 15 y	0 to < 12 m	1 to < 5 y	5 to < 15 y	0 to < 12 m	1 to < 5 y	5 to < 15 y
Presence of nurse within staff	2.53	3.17	N/A	19,426,468.17	2.81	0.75	0.63	0.16**	0.42
	(0.28–22.45)	(0.34–29.55)	N/A	N/A	(0.13–60.08)	(0.05–10.60)	(0.16–2.50)	(0.04–0.74)	(0.09–2.13)
Presence of health technician within staff	0.09**	0.14*	N/A	N/A	0.72	0.85	2.65	11.03**	0.56
	(0.01–0.86)	(0.01–1.36)	N/A	N/A	(0.02–25.41)	(0.05–14.97)	(0.46–15.32)	(1.65–73.63)	(0.07–4.54)
Staff dedicated to data management	0.01***	0.06*	N/A	N/A	0.23	0.52	4.34	0.81	0.17
	(0.00–0.30)	(0.00–1.12)	N/A	N/A	(0.01–10.69)	(0.03–9.13)	(0.51–36.82)	(0.07–9.64)	(0.01–2.10)
Staff trained in data management	8.35	6.01*	2.29e + 14	2.80	0.09	0.50	0.63	1.63	2.87
	(0.61–115.02)	(0.80–45.08)	(0.00–.)	(0.25–31.60)	(0.00–2.92)	(0.06–4.54)	(0.16–2.39)	(0.32–8.43)	(0.56–14.32)
New data collection form and reporting requirements	5.66*	3.97	24.28*	0.26	1.77	1.67	1.12	0.49	0.40
	(0.82–39.20)	(0.67–23.60)	(0.75–791.45)	(0.02–3.33)	(0.13–24.39)	(0.27–10.36)	(0.32–3.93)	(0.12–2.30)	(0.09–1.77)
Stock-outs of registers	7.25	1.95	3.72e+13	8.47	2.13	1.56	0.22*	7.82**	7.32**
	(0.65–80.61)	(0.22–17.06)	(0.00–.)	(0.43–166.86)	(0.08–54.52)	(0.16–14.95)	(0.04–1.23)	(1.20–50.78)	(1.14–47.23)
Stock-outs of malaria data collection forms	10.34***	7.84**	4,545,891.63	0.27	0.36	1.33	0.09***	0.70	2.36
	(1.20–53.49)	(1.24–49.54)	(0.00–.)	(0.02–3.09)	(0.02–7.60)	(0.09–18.68)	(0.02–0.37)	(0.13–3.71)	(0.47–11.81)
Stock-outs of HMIS forms	0.03**	0.03**	N/A	0.12	2.15	0.08*	1.92	0.11*	0.04***
	(0.00–0.73)	(0.00–0.44)	N/A	(0.00–2.33)	(0.03–139.80)	(0.01–1.44)	(0.27–13.74)	(0.01–1.08)	(0.00–0.46)
Rainy season	1.60	10.57***	6.64**	0.77	2.41	2.35	3.23***	2.88**	4.47**
	(0.62–4.15)	(2.32–48.19)	(1.38–32.04)	(0.26–2.32)	(0.60–9.72)	(0.69–8.03)	(1.38–7.54)	(1.15–7.19)	(1.43–13.96)
Stock-outs of RDT	0.10**	1.12	N/A	0.16	0.10*	1.97	0.92	1.32	0.54
	(0.014–0.78)	(0.21–6.01)	N/A	(0.01–3.79)	(0.01–1.31)	(0.30–13.01)	(0.25–3.38)	(0.26–6.54)	(0.10–2.82)

***p < 0.01, **p < 0.05, * p < 0.10

There was a lower odds of inaccurate reporting of suspected malaria cases among children 1 to < 5 years (0.10, 95% CI 0.01–1.31) in months with stock-outs RDTs, as there was no need to test cases (Table 3).

Stock-outs of HMIS forms were associated with the lower odds of inaccurate reporting confirmed malaria cases among children 1 to < 15 years (0.11, 95% CI 0.01–1.08 for children 1 to < 5 years; 0.04, 95% CI 0.00–0.46 for 5 to < 15 years) since staff may cope with using simplified self-adapted forms. In addition, the stock-outs of malaria data collection forms were associated with the lower odds of inaccuracy in reporting confirmed of simple malaria cases among children aged 0 to < 12 months (0.09, 95% CI 0.02–0.37). The presence of a nurse among clinic staff was associated with lower odds of inaccurate data for children 1 to < 5 years (0.16, 95% CI 0.04–0.74) whereas we found a higher odds of inaccurate reporting in clinics with a health technician among their staff (11.03, 95% CI 1.65–73.63). In addition, stock-outs of registers and the rainy season were associated with higher odds of inaccurate reporting malaria confirmed simple cases in HMIS (7.82, 95% CI 1.20–50.78 for children 1 to < 5 years; 7.32, 95% CI 1.14–47.23 for children 5 to < 15 years and 3.23, 95% CI 1.38–7.54 for children 0 to < 12 months; 2.88, 95% CI 1.15–7.19; 4.47 for children 1 to < 5 years, 95% CI 1.43–13.96 for children 5 to < 15 years) (Table 3).

## Discussion

Routine HMIS data are a readily-accessible resource for assessing the impact of public health interventions such as SMC. This study is the first to describe these data and the characteristics of clinics in a Chadian setting, and to attempt to assess current gaps in quality of data submitted for selected malaria indicators and identify factors influencing data accuracy.

We found a high level of completeness of data at the district and health centre level. This can be attributed to effective communication between the NMCP and health centre managers in case data for a specific month is missing. However, informal discussion with health centre staff shows the possibility of incorrect data declaration in case of delays in reporting, which means completeness does not necessarily lead to quality. In addition, in case of stock-outs of tools for data collection, health centres adapt by improvising tools which sometimes do not integrate disaggregated indicators as in the original tools. Thus as per health centre characteristics in 2018, efforts should be made to ensure that the 18.75% and 12.5% of facilities with stock-outs receive sufficient numbers of forms on time to improve accuracy and avoid staff having to improvise forms, which can lead to errors.

Similarly, accuracy of data was higher for data at district-level while it was low at health centre level for all indicators, which can be considered as a consequence of using paper-based forms [9]. Similar findings have been reported elsewhere [10]; authors have highlighted that the primary point of departure for accurate data transfer is during the tallying and collation process at the facility level, between the registers and routine monthly reports. In addition, other authors report an association between low accuracy at health facility level and workload due to multiple registers and tally sheets that need to be collated, summarized and sent to the next level [11]. This is particularly true for Chad where there are at least three tools at the health centre level for malaria data collection. This result highlighted the importance of an integrated system for data collection for all stakeholders.

Our results show a positive association between inaccuracy in data reporting in HMIS and the rainy season spanning July–October, when malaria transmission and caseload are highest [30]. In addition, half of health centres surveyed reported that they are understaffed, which is coherent with data at the national level [31] and in other sub-Saharan African countries [12, 19]. In addition, nine health centres experienced epidemics during the study period. These incidents can divert attention of data managers during data validation since health staff are focussed on its management. Other authors have also highlighted the role of organizational factors, including workload and training, in determining HMIS data quality [12]. During the rainy season, health centres may also over-report malaria cases to show to partners and the Ministry of Health the necessity of continued investment in malaria control, as reported elsewhere [19]. This situation is particularly relevant since the presence of a health technicians is significantly associated with lower odds of inaccurate reporting of confirmed malaria case among children aged 1 to < 5 years. On the contrary, the presence of a health technician is significantly associated with lower odds of inaccurate reporting of confirmed malaria cases among children aged 0 to < 12 months in HMIS. Another study in Ethiopia [19] has also reported lower odds of data reporting accuracy in clinics with a health technician among staff, which may reflect the positive effect of training, knowledge of importance of data and clinical experience on data quality as mentioned elsewhere [13, 19]. Contrary to findings from a study in Malawi [32], presence of dedicated staff for data management at health facilities was associated with higher odds of data accuracy. Presence of staff dedicated exclusively to data management can improve data quality over time.

The results show a negative association between stock-outs of HMIS forms and inaccurate reporting of attendance for malaria. This can be understood by the fact that health facilities may cope with stock-outs by improvising their own forms which may be easier to complete than the original which has a small font size. In addition, to ease their work, the health facility staff may not have disaggregated the malaria cases by gender, as required for the original HMIS form. Similarly, our results show an association between higher odds of inaccurate attendance reporting and changes to the malaria data collection form which disaggregated data by age group and gender. This new requirement has been criticized by health centre managers as being difficult to implement. Others authors have also highlighted the design of data collection tools as a reason for poor quality data [12–14]. Others still have gone further, mentioning difficulties in differentiating suspected and confirmed malaria cases which may contribute to misreporting of malaria cases [15, 16]. To tackle the problem of stock-outs of forms, multiple data collection tools for different partners and errors related to paper based form, an integrated web-based health management information system may provide a solution.

### Limitations and strengths

The sample, which included all health centres in Massaguet, may not be representative of all facilities nationally. We used data from January–December 2018, which can represent a limitation since the study’s findings may not be generalizable over time. However, the reasons for chosen this period were: (i) at the time of ethical approval and the implementation of the study, the HMIS data for 2019 were not yet validated; (ii) 2018 was a relatively stable year which can be a good baseline against which any potential intervention to improve data quality may be compared; (iii) the time needed for data extraction; (iv) the time required for additional approval from the national ethics committee; and (v) the need of evidence for decision-making related to the SMC extension, such that we did not consider data after 2018. We did not assess the impact of implementation of free-of-charge care, which may have increased staff workload [33], and resulting changes in the data collation process on data accuracy. While this study’s findings may inform future research on HMIS data elements in Chad and other countries, the degree to which our findings are generalizable to other comparable settings is uncertain. However the fact that all type of facilities (public, confessional and community) reporting data to the HMIS were included is valuable and we are confident that the findings of this study can help with understanding the level of agreement between HMIS data and facility source documents’ records for the indicators considered. In terms of theory, this study highlights the usability of verification factors as tool for assessing internal consistency of data, particularly in the Chadian context.

## Conclusions

We found a high level of completeness of data at the district and health centre level while accuracy was low for all indicators at the health centre level. Low workload, presence of dedicated staff for data management, training, and the availability of tools for data collection, were significantly associated with the accuracy of HMIS data. Upgrading from paper-based forms to a web-based health management information system may provide a solution for reducing errors in the reporting of health indicators, and improve the accuracy of data and its potential utility for decision-making on key malaria control interventions and their impact evaluations. Such an initiative would be contingent on sufficient funding and the provision of computers and infrastructure such as electricity and internet connections. Further efforts are also required to ease the workload of health facility personnel. Although current HMIS data can be readily used for assessing the impact of public health interventions, researchers should consider the quality of HMIS data and the potential need to adjust analyses or contextualize results given the greater degree of inaccuracy during the rainy season. Future research can assess other dimensions of data governance.

## Supplementary Information

**Additional file 1: Table S4**. Characteristics of health centres surveyed

**Additional file 2**. Questionnaire for register data extraction

**Additional file 3**. CODEBOOK for form: Chad_clinical_data_quality_audit_Clinic_questionnaire

## Data Availability

The datasets generated during and/or analyzed during the current study are available from the corresponding author on reasonable request.

## Abbreviations

SDSIS: Department of Statistics and Health Information
HMIS: Health Management Information System
NMCP: National Malaria Control Program
SMC: Seasonal Malaria Chemoprevention
VF: Verification factor
WHO: World Health Organization
