# Supplementary material for: Health management information system (HMIS) data quality and associated factors in Massaguet district, Chad
Source: BMC Med Inform Decis Mak. 2021 Nov 22;21:326. doi: 10.1186/s12911-021-01684-7 (PMC8609810; doi:10.1186/s12911-021-01684-7)
Supplement: Supplementary file 1 — Additional file 1: Table S4. Characteristics of health centres surveyed [file 12911_2021_1684_MOESM1_ESM.docx]

**Title: Health Management Information System (HMIS) data quality and associated factors in Massaguet district, Chad.**

**Short title: HMIS data quality and associated factors in Chad**

**Authors:** Azoukalné Moukénet, Monica Anna de Cola, Charlotte Ward, Beakgoubé Honoré, Kevin Baker, Laura Donovan, Laoukolé Jean, Sol Richardson

**Additional file 1**

Table 4 : Characteristics of health centres surveyed

| Variables | | Freq. | Percent | Mean (CI) |
| --- | --- | --- | --- | --- |
| Location (N=16) | Rural | 13 | 81.25 |  |
|  | Peri urban | 2 | 12.50 |  |
|  | Urban | 1 | 6.25 |  |
| Electricity supply (N=16) | Yes | 13 | 81.25 |  |
| Consultation and treatment room separate (N=16) | Yes | 15 | 93.75 |  |
| Observation bed(s) (N=16) | Yes | 12 | 75.00 |  |
| Functioning community platform (N=16) | Yes | 13 | 86.67 |  |
| Presence of nurse within staff (N=16) | Yes | 7 | 43.75 |  |
| Presence of midwife within staff (N=16) | Yes | 7 | 43.75 |  |
| Presence of technical agent within staff (N=16) | Yes | 15 | 93.75 |  |
| Presence of technical health agent within staff (N=16) | Yes | 10 | 62.50 |  |
| Water supply (N=16) | No delivered | 6 | 37.50 |  |
|  | Shared | 3 | 18.75 |  |
|  | Yes own | 7 | 43.75 |  |
| Vehicle for patient transport (N=16) | No vehicle | 2 | 12.50 |  |
|  | Shared district vehicle | 13 | 81.25 |  |
|  | Own vehicle | 1 | 6.25 |  |
| Opinion on workload in health center (N=16) | Severely understaffed | 1 | 6.25 |  |
|  | Somewhat understaffed | 7 | 43.75 |  |
|  | Generally sufficiently staffed | 8 | 50.00 |  |
| Strikes occurred disturbed facility operations (N=16) | Yes | 5 | 31.25 |  |
| Flow occurred disturbed facility operations (N=16) | Yes | 3 | 18.75 |  |
| Epidemic occurred disturbed facility operations (N=16) | Yes | 2 | 12.50 |  |
| Stock-out rdt test (N=16) | Yes | 9 | 56.25 |  |
| Inspection by government or NMCP (N=16) | Yes | 9 | 56.25 |  |
| Stock-out of antimalarials (N=16) | Yes | 9 | 56.25 |  |
| Stock-out of registers (N=16) | Yes | 6 | 37.50 |  |
| Stock-out of HMIS forms (N=16) | Yes | 2 | 12.50 |  |
| Stock out of malaria data record forms (N=16) | Yes | 3 | 18.75 |  |
| Arrears in payment of malaria data incentive (N=16) | Yes | 12 | 75.00 |  |
| Presence of staff dedicated to data management (N=16) | Yes | 8 | 50.00 |  |
| Data management staff received training (N=16) | Yes | 9 | 56.25 |  |
| Data managerand health center in same region (N=16) | Yes | 4 | 25.00 |  |
| New data collection form and reporting requirements, issued 2018 (N=16) | Yes | 4 | 25.00 |  |
| Staff trained in RDT management (N=16) | Yes | 12 | 75.00 |  |
| Population (N=16) |  |  |  | 10294.23 |
|  |  |  |  | (10239.15 – 10349.53) |
| Number of observation beds (N=16) |  |  |  | 2.58 |
|  |  |  |  | (1.76 – 3.67) |
| Number of staff all types (N=16) |  |  |  | 4.94 |
|  |  |  |  | (3.91 – 6.15) |
| Number of volunteer staff paid by community (N=16) |  |  |  | 2.63 |
|  |  |  |  | (1.89 – 3.55) |
| Number of days of stock-outs of RDT (N=192) |  |  |  | 16.53 |
|  |  |  |  | (14.54 – 18.72) |
| Number of days of stock-outs of antimalarials (N=192) |  |  |  | 21.13 |
|  |  |  |  | (18.93 – 23.50) |
| Number of days of stock-outs of of registers (N=192) |  |  |  | 30.32 |
|  |  |  |  | (27.89 – 32.90) |
| Number of days of stock-outs of HMIS forms (N=192) |  |  |  | 26.83 |
|  |  |  |  | (22.85 -31.31) |
| Number of days of stock-outs of malaria data collection forms (N=192) |  |  |  | 23.13 |
|  |  |  |  | (19.91 – 26.71) |
| Number of days of arrears malaria in payment of malaria data incentive (N=192) |  |  |  | 29.24 |
|  |  |  |  | (28.37 – 30.12) |
| Training duration (N=16) |  |  |  | 2.75 |
|  |  |  |  | (1.72 – 4.16) |
| Data manger’s number of years’ experience in data management (N=16) |  |  |  | 7.44 |
|  |  |  |  | (5.77 – 9.45) |
