# Supplementary material for: Health management information system (HMIS) data quality and associated factors in Massaguet district, Chad
Source: BMC Med Inform Decis Mak. 2021 Nov 22;21:326. doi: 10.1186/s12911-021-01684-7 (PMC8609810; doi:10.1186/s12911-021-01684-7)
Supplement: Supplementary file 2 — Additional file 2. Questionnaire for register data extraction [file 12911_2021_1684_MOESM2_ESM.docx]

**Title: Health Management Information System (HMIS) data quality and associated factors in Massaguet district, Chad.**

**Short title: HMIS data quality and associated factors in Chad**

**Authors:** Azoukalné Moukénet, Monica Anna de Cola, Charlotte Ward, Beakgoubé Honoré, Kevin Baker, Laura Donovan, Laoukolé Jean, Sol Richardson

**Additional File 2:**

**QUESTIONNAIRE FOR REGISTER DATA EXTRACTION**

**CODEBOOK for form: Research_HF_Form_Chad**

| Description |  |
| --- | --- |
| Created | 2019-12-28 10:55:17.0 |
| Last edited | 2020-01-07 10:20:38.0 |
| Owner | [intentionally left blank] |
| Tags |  |
| GPS stamp | yes |
| Questions | 10 |

**1. FORMULAIRE DE COLLECTE DE DONNEES DES REGISTRES CENTRES DE SANTE 2018 [FORM FOR DATA COLLECTION FROM CLINICAL REGISTER 2018]**

**2.Centre de Santé [Health centre]**

Question Type= dropdown (cascading)

Data Field Name : health_centre

Choose one response:

- Abou Halime (Abou_Halime)

- Absouf (Absouf)

- Afrouk (Afrouk)

- Amdedoua (Amdedoua)

- Amdourman (Amdourman)

- Birbarka (Birbarka)

- Brekate (Brekate)

- Chawa (Chawa)

- Djermaya (Djermaya)

- Farcha attere (Farcha_attere)

- Filey (Filey)

- Hawich (Hawich)

- Karme (Karme)

- Massaguet urbain (Massaguet_urbain)

- Naala (Naala)

- N'Djamena Fara (N_Djamena_Fara)

**3.Mois [Month]**

Question Type= dropdown

Data Field Name : month

Choose one response:

- Janvier (January)

- Février (February)

- Mars (March)

- Avril (April)

- Mai (May)

- Juin (June)

- Juillet (July)

- Août (August)

- Septembre (September)

- Octobre (October)

- Novembre (November)

- Décembre (December)

**4.Population du centre de santé [Population in the catchment area of the health centre]**

Question Type= numeric

Data Field Name : number_people_catchment_area

Min 1

Max

**5. Informations sur les patients: ouvrir autant de sous formulaire qu'il y a de patient de moins de 15 ans! [Information about patients: please add many forms, as there are patient under 15 years old!]**

**6.Sous formulaire patient du registre [Sub-form patient in the register]**

Question Type= Subform

Data Field Name : subform_patient_registry

Subform Name : subform_research_Chad_patient

Subform Keyword : subform_patient_registry_HF

**7. Information sur le stock des intrants du centre [Information on stock of input in health centre]**

**8.Y a-t-il eu de rupture de stock de TDR dans le mois? [Have you had a stock-out of RDT test this month]**

Question Type= dropdown

Data Field Name : rdtstockout_happened

Choose one response:

- Oui (Yes)

- Non (No) If this response, jump to 10

**9.Quel est le nombre cumulé de jour rupture de stock de TDR dans le mois? [What is the duration of stock out of RDT in this month]**

Question Type= numeric

Data Field Name : Number_dayrdt_stockout

Min 1

Max 31

**10. Fin de collecte de données du mois pour le centre de santé choisi [End of data collection for the month selected for this health centre]**

**Subform: subform_research_Chad_patient**

| Description |  |
| --- | --- |
| Created | 2019-12-28 11:55:37.0 |
| Last edited | 2020-01-07 10:17:03.0 |
| Owner | [intentionally left blank] |
| Tags |  |
| GPS stamp | yes |
| Questions | 20 |

**1. Informations socio-démographiques du patient [Socio-demographic information on the patient]**

**2.Numéro du patient sur le registre [Id of patient into the register]**

Question Type= numeric

Data Field Name : Patient_num

**3.Patient de moins de 5 ans? [Patient under 5 years old ?]**

Question Type= dropdown

Data Field Name : which_age

Choose one response:

- Oui (yes) If this response, jump to 5

- Non (no)

**4.Age du patient en années [Age of patient in years]**

Question Type= numeric

Data Field Name : age_years

Min 5

Max 14

**5.Age du patient en mois (si le patient a moins de 5 ans) [Age of patient in month (if the patient is under 5 years old]**

Question Type= numeric

Data Field Name : age_months

Min 0

Max

**6.Sexe du patient [Gender of patient]**

Question Type= radio

Data Field Name : gender

Choose one response:

- Féminin (female)

- Masculin (male)

**7.Zone de provenance du patient [Area of patient location]**

Question Type= dropdown

Data Field Name : area

Choose one response:

- Zone A (area_A)

- Zone B (area_B)

- Nomade (Nomad)

- Hors zone (out_area)

**8.Description clinique de problème de santé [Clinic description of the health problem]**

Question Type= checkbox

Data Field Name : clinical_symptom

Choose all that apply:

- Fièvre (fever)

- Autres (others)

**9. Problèmes de santé [Health issue]**

**10.1er problème de santé [1st main health problem]**

Question Type= Text

Data Field Name : health_pbme1

**11.2e problème de santé [2nd main health problem]**

Question Type= Text

Data Field Name : health_pbme2

**12.3e problème de santé [3rd main health problem]**

Question Type= Text

Data Field Name : health_pbme3

**13.Test du paludisme (TDR et test microscopique) [Malaria test (RDT of microscopy)]**

Question Type= checkbox

Data Field Name : malaria_test

Choose all that apply:

- TDR + (positive_rdt)

- TDR - (negative_rdt)

- GE + (positive_microbio)

- GE - (negative_microbio)

- Absence test (no_test)

**14. Traitement et conduite à tenir [Treatment and advice prescribed]**

**15.1er traitement [1^st^ treatment]**

Question Type= Text

Data Field Name : treatment1

**16.2e traitement [2^nd^ treatment]**

Question Type= Text

Data Field Name : treatment2

**17.3e traitement [3^rd^ treatment]**

Question Type= Text

Data Field Name : treatment3

**18. Issue du patient [Patient outcome]**

**19.Issue du patient [Patient outcome]**

Question Type= dropdown

Data Field Name : patient_outcome

Choose one response:

- Référé (refere)

- Décédé (death)

- Hospitalisation (hospitalization)

- Autres (others)

**20. Prière passer au patient suivant [Please go to the next patient]**
