# Supplementary material for: Health management information system (HMIS) data quality and associated factors in Massaguet district, Chad
Source: BMC Med Inform Decis Mak. 2021 Nov 22;21:326. doi: 10.1186/s12911-021-01684-7 (PMC8609810; doi:10.1186/s12911-021-01684-7)
Supplement: Supplementary file 3 — Additional file 3. CODEBOOK for form: Chad_clinical_data_quality_audit_Clinic_questionnaire [file 12911_2021_1684_MOESM3_ESM.docx]

**Title: Health Management Information System (HMIS) data quality and associated factors in Massaguet district, Chad.**

**Short title: HMIS data quality and associated factors in Chad**

**Authors:** Azoukalné Moukénet, Monica Anna de Cola, Charlotte Ward, Beakgoubé Honoré, Kevin Baker, Laura Donovan, Laoukolé Jean, Sol Richardson

**Additional file 3:**

**CODEBOOK for form: Chad_clinical_data_quality_audit_Clinic_questionnaire**

| Description |  |
| --- | --- |
| Created | 2020-03-05 14:52:23.0 |
| Last edited | 2020-03-19 08:37:16.0 |
| Owner | [intentionally left blank] |
| Tags |  |
| GPS stamp | yes |
| Questions | 165 |

**1.Aire de Santé [Health Centre]**

Question Type= dropdown (cascading)

Data Field Name : CS

Choose one response:

- Abou Halimé (Abou_Halime)

- Absouf (Absouf)

- Afrouk (Afrouk)

- Amdedoua (Amdedoua)

- Amdourman (Amdourman)

- Birbarka (Birbarka)

- Brekate (Brekate)

- Chawa (Chawa)

- Djermaya (Djermaya)

- Farcha attere (Farcha_attere)

- Filey (Filey)

- Hawich (Hawich)

- Karme (Karme)

- Massaguet urbain (Massaguet_urbain)

- Naala (Naala)

- N'djamena fara (Ndjamena_fara)

**2.Date [Date]**

Question Type= date

Data Field Name : Date_of_questionnaire

**3.Personne interviewee [Respondent to survey]**

Question Type= radio

Data Field Name : Person_interviewed

Choose one response:

- Responsable Centre de santé (Clinic_manager) If this response, jump to 6

- Autre personnel (Other_staff_member)

**4.Si autre personnel, prière spécifier leur fonction [If other persons, please mentionned his role]**

Question Type= Text

Data Field Name : If_other_staff_role

**5.Si autre personnel, pourquoi le responsable de centre de santé n'est pas disponible? [If other personnel, why the health center manager were absent?]**

Question Type= Text

Data Field Name : other_why_no_clinic_manager

**6.Ce centre de santé se trouve en milieu urbain ou rural? [This health center is located in urban or rural area?]**

Question Type= radio

Data Field Name : clinic_located_urban_rural

Choose one response:

- Urbain (Urban)

- Peri-urbain (Peri_urban)

- Rural (Rural)

**7.A quelle type de structure appartient ce centre de santé? [To which organization is this health center belong?]**

Question Type= radio

Data Field Name : Type_provider_manages_clinic

Choose one response:

- Structure publique (Public_organisation) If this response, jump to 9

- Structure privée (Private_organisation) If this response, jump to 9

- Structure confessionnelle (Religious_organisation) If this response, jump to 9

- Centre de santé ONG ou caritative (Secular_charity_NGO) If this response, jump to 9

- Autre (Other)

**8.Si autre, prière spécifier [If other, please mentioned]**

Question Type= Text

Data Field Name : If_other_specify

**9.Veuillez estimer le nombre de personne vivant dans cette zone de responsabilité [Please estimate the population living the catchment area]**

Question Type= numeric

Data Field Name : Number_people_served_clinic

**10.Le centre a-t-il de l'électricité? [The health center have access to electricity supply]**

Question Type= radio

Data Field Name : Clinic_electricity_supply

Choose one response:

- Oui, connecté au réseau électrique (Yes_power_grid)

- Oui, fourni par son propre générateur (Yes_own_generator)

- Non (No)

**11.Le centre de santé a-t-il se propre source d'eau? [The health centre have access to water supply?]**

Question Type= radio

Data Field Name : Clinic_water_supply

Choose one response:

- Oui, le centre de santé a sa propre source d’eau (Yes_own_water_supply)

- Il y a une source d’eau près du centre de santé mais elle est partagée avec d’autres structures (Water_supply_shared)

- Non, l'eau est livrée (No_water_delivered)

**12.Le centre a-t-il une ligne téléphonique dédiée pour le service? [The health center has a dedicated phone ?]**

Question Type= radio

Data Field Name : Clinic_dedicated_phone

Choose one response:

- Oui, le centre possède une ligne téléphonique ou téléphone portable dédiée au service (Yes_own_landline_mobile)

- Non, mais le téléphone privé du personnel utilisé pour le service (No_staff_private_phone)

- Non (No)

**13.Le centre de santé a-t-il son propre véhicule dédié au transport de patients? [The health centre has a vehicle for patient transport ?]**

Question Type= radio

Data Field Name : Clinic_vehicle_patient_transp

Choose one response:

- Oui, le centre de santé dispose d'un véhicule dédié au transport de patients (Yes_own_vehicle_patient_transport)

- Non, mais le centre a accès à un véhicule utilisé en partage avec d'autres centre de santé ou le district (No_but_access_vehicle_shared_clinic_district)

- Non (No)

**14.Le centre dispose t-il des toilettes? [The health centre has a toilet facility?]**

Question Type= radio

Data Field Name : Clinic_toilet_facility

Choose one response:

- Oui (Yes)

- Non (il n'y a pas de toilette ou l'utilisation de toilettes est en partage avec d'autres structures voisines) (No_no_toilet_or_shared)

**15.Le centre de santé a-t-il un système de gestion de déchets cliniques distinct de déchets ordinaires? [The health centre has separate clinic waste ?]**

Question Type= radio

Data Field Name : Clinic_separate_clinic_waste_g

Choose one response:

- Oui (Yes)

- Non (No)

**16.Le centre de santé dispose-t-il d’un incinérateur (pour éliminer les déchets) ? [The health centre has an incinarator ?]**

Question Type= radio

Data Field Name : waste_incinator

Choose one response:

- Oui (Yes)

- Non (No)

**17.Le centre de santé dispose-t-il d’un dispositif saine de traitement des eaux usées ? [The health centre has a safe waste water ?]**

Question Type= radio

Data Field Name : Clinic_safe_wastewater

Choose one response:

- Oui (évacuation dans un puisoir, utilisation d’un système de traitement ou d’assainissement sur place, épuration sur place) (Yes_sewer_onsite_treatment_sanitation_system_onsite_desinfection)

- Non (évacuation des eaux usées dans les eaux locales ou sur le sol) (No_direct_discharge_local_water_bodies_fields)

**18.Le centre de santé a-t-il un local de consultation préservant l’intimité du patient (ex :salle de consultation en séparé de la salle d’attente) [The health centre has a private patient consultation room ?]**

Question Type= radio

Data Field Name : Clinic_private_patient_consult

Choose one response:

- Oui (Yes)

- Non (No)

**19.Le centre de santé a-t-il une salle de soins dédiée et séparée de la salle de consultation? [The health centre has a dedicated consultation room separate from treatment room?]**

Question Type= radio

Data Field Name : Clinic_consult_treatment_room

Choose one response:

- Oui (Yes)

- Non (No)

**20.Le centre de santé dispose-t-il d’une salle de pharmacie ? [The health centre has a pharmacy room?]**

Question Type= radio

Data Field Name : Clinic_pharmacy_room

Choose one response:

- Oui (Yes)

- Non (No)

**21.Le centre de santé a-t-il un dispositif de lavage de mains ? [The health centre has a hand washing facility?]**

Question Type= radio

Data Field Name : Clinic_hand_washin_facility

Choose one response:

- Oui, le dispositif de lavage de main du personnel du centre est séparé de celui des patients (Yes_separate_hand_wash_staff_patients)

- Oui, mais le dispositif de lavage de main est partagé entre le personnel du centre et les patients (Yes_shared_staff_patients)

- Non (No)

**22.Le centre de santé dispose-t-il des lits d’observation de patients ? [The health centre has an observation bed for patient ?]**

Question Type= radio

Data Field Name : Clinic_observation_bed_patient

Choose one response:

- Oui (Yes)

- Non (No) If this response, jump to 25

**23.Si oui, combien? [If yes, how many?]**

Question Type= numeric

Data Field Name : Yes_number

**24.Si oui, les lits d’observation sont-ils dans une salle dédiée à l’observation ? [If yes, are the observation beds in a dedicated room for observation?]**

Question Type= radio

Data Field Name : Yes_obervation_beds_dedicated

Choose one response:

- Oui (Yes)

- Non (No)

**25.Le centre de santé a-t-il un système défini pour la référence de cas complexes (sévères) au niveau secondaire ou hôpital de district ? [The health centre has a system for refeering severe cases to a district hospital?]**

Question Type= radio

Data Field Name : Clinic_system_referring

Choose one response:

- Oui (Yes)

- Non (No)

**26.Le centre de santé dispose-t-il d’un ordinateur ? [The health centre has a computer?]**

Question Type= radio

Data Field Name : Clinic_access_computer

Choose one response:

- Oui, il y a un ordinateur dédié ou accès à un ordinateur privé pour le service (Yes_dedicated_computer_private_computer)

- Non (No)

**27.Le centre de santé a-t-il une plateforme communautaire (COGES) fonctionnelle ? [The health centre [The health centre has a functionnal community platform?]**

Question Type= radio

Data Field Name : Clinic_funct_community_platfor

Choose one response:

- Oui (Yes)

- Non (No)

**28.Combien de personnel toutes catégories (incluant les volontaires et le personnel sans qualification clinique) travaille dans ce centre de santé (le mois actuel) [How many personnel all types work in this health centre (the current month) ?]**

Question Type= numeric

Data Field Name : Number_staff_all_types_month

**29.Selon les critères d’opérationnalité du MSP, lesquels de catégories professionnelles suivant dispose votre centre ? [In relation to the criteria for operationality of the MOH, which of the below health staff categories work in your health centre?]**

Question Type= checkbox

Data Field Name : Categories_staff_work

Choose all that apply:

- Infirmier Diplômé d'Etat (Nurse)

- Sage-Femme Diplômée d'Etat (Midwife)

- Agent Technique de Santé (Nurse_assistant)

- Technicien de surface (Cleaner)

**30.Parmi l’équipe du centre de santé, y-a-t-il du personnel volontaire ou payé par la communauté ? [Among the staff of health centre are there volunteers or paid by the community?]**

Question Type= radio

Data Field Name : Staff_volunteers_salaries_comm

Choose one response:

- Oui (Yes)

- Non (No) If this response, jump to 33

**31.Si oui, décrire la situation [If yes, describe the situation]**

Question Type= Text

Data Field Name : Describe_situation

**32.Mentionner le nombre [Mention the number]**

Question Type= numeric

Data Field Name : Number_volunteers_salaries_com

**33.Le responsable du centre de santé est-il originaire de la province dont se trouve le centre ? [Is the health centre responsible originate of the province where is locate the health centre?]**

Question Type= radio

Data Field Name : Clinic_manager_region_clinic

Choose one response:

- Oui (Yes)

- Non (No)

**34.Selon vous, laquelle des assertions suivantes est vrai (choisir une) ? Le centre de santé est : [According to you, which of the following declarations is a truth? Your health centre is:]**

Question Type= radio

Data Field Name : Opinion_truth_clinic

Choose one response:

- Presqu’à tout moment, le personnel est en gravement en sous-effectif pour répondre à la demande (Severely_understaffed_demand_most_situation)

- Le personnel est presqu’en sous-effectif et a du mal à répondre à la demande en période de forte affluence (Somewhat_understaffed_demand_busy_periods)

- Globalement le personnel est suffisant pour répondre à la demande et même dans la plupart de périodes d’affluence (Generally_sufficiently_staffed_demand_busy_periods)

**35.Selon vous, les conditions de travail du personnel affectent-elles négativement les activités du centre de santé et les soins effectués ? [According to you, the work condition negatively the activities and health provide in the health centre]**

Question Type= radio

Data Field Name : Opinion_truth_staff_problems

Choose one response:

- Oui (Yes)

- Non (No) If this response, jump to 37

**36.Si oui, veuillez décrire la situation [If yes, please describe the situation]**

Question Type= Text

Data Field Name : Describe_situation_staff_probl

**37.Le centre de santé a-t-il un personnel spécialement dédié à la gestion de données (pour l’enregistrement des cas de paludisme) ? [The health centre has a special staff dedicated to data management (for recording malaria cases)?]**

Question Type= radio

Data Field Name : Clinic_staff_dedicated_data

Choose one response:

- Oui, et c’est sa tâche principale (Yes_main_role) If this response, jump to 39

- Oui, mais c’est une de ses tâches secondaires (mentionné dans sa description de tâches) (Yes_secondary_roles)

- Non (aucun personnel spécifique n’est responsable de gestion des données) (No_no_specific_staff) If this response, jump to 39

**38.Si oui, mais c’est une de ses tâches secondaires, veuillez lister les autres tâches effectuées [If yes, it is a secondary task, please list the other tasks]**

Question Type= Text

Data Field Name : List_other_tasks_if_secondary

**39.Le personnel responsable de la gestion des données a-t-il reçu une formation sur la collecte, l’enregistrement et ou la gestion de données ? [The staff responsible of data management received a training on the data recording and/or data management?]**

Question Type= radio

Data Field Name : Clinic_staff_training_data

Choose one response:

- Oui (Yes)

- Non (No) If this response, jump to 45

**40.Si oui, quelle type de formation a-t-il reçu? [If yes, which type of training did he received?]**

Question Type= Text

Data Field Name : Type_training_received

**41.Si oui, qui a facilité cette formation? [If yes, who facilitated that training?]**

Question Type= Text

Data Field Name : Training_facilitator

**42.Si oui, quelle était la durée de la dernière formation reçue par la personne ? (en jours) [If yes, what is the duration of the last training that person attained?]**

Question Type= numeric

Data Field Name : Training_duration_days

**43.Si oui, depuis quand cette personne a reçu la dernière formation ? (en années) [If yes, how long that person received the last training?]**

Question Type= numeric

Data Field Name : How_long_training_years

**44.Si oui, combien d’années d’expérience a cette personne dans la collecte, l’enregistrement et ou la gestion de données ? [If yes, how many years of experience in data collection, recording and/or management has that person?]**

Question Type= numeric

Data Field Name : Number_years_experience_data

**45.La personne en charge de la collecte, l’enregistrement et ou la gestion de données est-elle originaire de la province où se trouve le centre de santé ? [The person responsible of data collection, recording and/or management is from the province where is locate the health centre?]**

Question Type= radio

Data Field Name : Responsible_data_region_clinic

Choose one response:

- Le responsable de centre de santé et la personne en charge de la collecte, l’enregistrement et ou la gestion de données sont les mêmes (clinic_manager_responsible_data_same)

- Oui (Yes)

- Non (No)

- N/A (NA)

**46.Durant l’année 2018, la prise en charge des cas du paludisme a-t-elle été affectée négativement par les grèves du personnel ? [During 2018, did the case management of malaria affected negatively by the personnel strikes?]**

Question Type= radio

Data Field Name : During_2018_malaria_strikes

Choose one response:

- Oui (Yes)

- Non (No) If this response, jump to 49

**47.Si oui, quels mois? (cocher les mois concernés) [If yes, which months? (please check all corresponding months)]**

Question Type= checkbox

Data Field Name : Which_month_strick

Choose all that apply:

- Janvier (January)

- Février (February)

- Mars (March)

- Avril (April)

- Mai (May)

- Juin (June)

- Juillet (July)

- Août (August)

- Septembre (September)

- Octobre (October)

- Novembre (November)

- Décembre (December)

**48.Durant l’année 2018, la prise en charge des cas du paludisme a-t-elle été affectée négativement par d'autres facteurs ou événements ? [During 2018, did the case management of malaria affected negatively by the others factors or events?]**

Question Type= radio

Data Field Name : During_2018_malaria_others_fac

Choose one response:

- Oui (Yes)

- Non (No) If this response, jump to 52

**49.Un des événements suivants s’est produit en 2018 ? (cocher tous les événements concernés) [One of the below events happened in 2018? (please check all corresponding events)]**

Question Type= checkbox

Data Field Name : Following_occur_2018

Choose all that apply:

- Inondation (Flooding) If this response, jump to 52

- Grand vol des biens du centre de santé (Major_theft_clinic_property) If this response, jump to 52

- Incident violent majeur ayant affecté le personnel (Major_violent_incident_staff) If this response, jump to 52

- Domage significatif sur les biens du centre de santé (ex: incendie, écroulement d’immeubles) (Damage_clinic_property) If this response, jump to 52

- Conflit communautaire (Community_violence) If this response, jump to 52

- Apparition d’épidémie (en dehors du paludisme, ex: méningite, rougeole, choléra) (Epidemic_disease_outbreak) If this response, jump to 52

- Autres facteurs ou événemets (Others_factors_events)

**50.Veuillez décrire tout autres facteurs ou événements qui s’est produits [Please describe all other factors or events happened]**

Question Type= Text

Data Field Name : Other_factors_events_occured

**51.Si oui, quels mois? (veuillez cocher tous les mois concernés par un des facteurs/événements mentionné) [If yes, which months ? (please check all months when one of the above factors / events happened)]**

Question Type= checkbox

Data Field Name : Which_month_other_fact_events

Choose all that apply:

- Janvier (January)

- Février (February)

- Mars (March)

- Avril (April)

- Mai (May)

- Juin (June)

- Juillet (July)

- Août (August)

- Septembre (September)

- Octobre (October)

- Novembre (November)

- Décembre (December)

**52.Durant l’année 2018, le centre de santé a-t-il été à mesure d’envoyer régulièrement les données du paludisme dans les cinq jours après chaque fin de mois ? [During 2018, the health centre was able to share the data on malaria within five days after each end of month?]**

Question Type= radio

Data Field Name : During_2018_malaria_data_sent

Choose one response:

- Oui (Yes) If this response, jump to 55

- Non (No)

**53.Si non, veuillez décrire pourquoi [If no, please describe why]**

Question Type= Text

Data Field Name : No_why_describe

**54.Si non, quels mois étaient concernés par le retard d’envoie de données au district ? (veuillez cocher tout les mois concernés) [If no, which months there were delays of sharing data with the disctrict?]**

Question Type= checkbox

Data Field Name : Which_month_data_sent_late

Choose all that apply:

- Janvier (January)

- Février (February)

- Mars (March)

- Avril (April)

- Mai (May)

- Juin (June)

- Juillet (July)

- Août (August)

- Septembre (September)

- Octobre (October)

- Novembre (November)

- Décembre (December)

**55.Durant l’année 2018, la nouvelle exigence de collecte de données réparties par genre vous a-t-elle crée de difficultés ? [In 2018, the requirement for fill malaria data by gender was a challenge for you?]**

Question Type= radio

Data Field Name : During_2018_new_requirement_pb

Choose one response:

- Oui (Yes)

- Non (No) If this response, jump to 57

**56.Si oui, veuillez décrire le (s) problème (s) [If yes, please describe which problems]**

Question Type= Text

Data Field Name : Yes_why_describe_requirement_p

**57.Durant l’année 2018, le centre de santé a-t-il effectué des tests microbiologiques (GE) dans un des mois ? [In 2018, the health centre has performed the microscopy test of malaria at least in one month?]**

Question Type= radio

Data Field Name : During_2018_stockout_microbio

Choose one response:

- Oui (Yes)

- Non (No) If this response, jump to 71

**58.A quels mois y a-t-il eu rupture de stock tests microbiologiques et pour combien de jours ? (veuillez cocher les mois concernés) [In which month there was a stock-out of microscopy test and how many days?]**

Question Type= checkbox

Data Field Name : Which_month_stockout_microbio

Choose all that apply:

- Janvier (January)

- Février (February)

- Mars (March)

- Avril (April)

- Mai (May)

- Juin (June)

- Juillet (July)

- Août (August)

- Septembre (September)

- Octobre (October)

- Novembre (November)

- Décembre (December)

**59.Nombre de jours de rupture de stock GE janvier [Number days of stock-out of microscopy test in January]**

Question Type= numeric

Data Field Name : Number_stockout_ge_jan

Min 0

Max 31

**60.Nombre de jours de rupture de stock GE février [Number days of stock-out of microscopy test in February]**

Question Type= numeric

Data Field Name : Number_stockout_ge_feb

Min 0

Max 28

**61.Nombre de jours de rupture de stock GE mars [Number days of stock-out of microscopy test in March]**

Question Type= numeric

Data Field Name : Number_stockout_ge_march

Min 0

Max 31

**62.Nombre de jours de rupture de stock GE avril [Number days of stock-out of microscopy test in April]**

Question Type= numeric

Data Field Name : Number_stockout_ge_april

Min 0

Max 30

**63.Nombre de jours de rupture de stock GE mai [Number days of stock-out of microscopy test in May]**

Question Type= numeric

Data Field Name : Number_stockout_ge_may

Min 0

Max 30

**64.Nombre de jours de rupture de stock GE juin [Number days of stock-out of microscopy test in June]**

Question Type= numeric

Data Field Name : Number_stockout_ge_june

Min 0

Max 31

**65.Nombre de jours de rupture de stock GE juillet [Number days of stock-out of microscopy test in July]**

Question Type= numeric

Data Field Name : Number_stockout_ge_july

Min 0

Max 30

**66.Nombre de jours de rupture de stock GE août [Number days of stock-out of microscopy test in August]**

Question Type= numeric

Data Field Name : Number_stockout_ge_august

Min 0

Max 31

**67.Nombre de jours de rupture de stock GE septembre [Number days of stock-out of microscopy test in September]**

Question Type= numeric

Data Field Name : Number_stockout_ge_sept

Min 0

Max 30

**68.Nombre de jours de rupture de stock GE octobre [Number days of stock-out of microscopy test in October]**

Question Type= numeric

Data Field Name : Number_stockout_ge_oct

Min 0

Max 31

**69.Nombre de jours de rupture de stock GE novembre [Number days of stock-out of microscopy test in November]**

Question Type= numeric

Data Field Name : Number_stockout_ge_nov

Min 0

Max 30

**70.Nombre de jours de rupture de stock GE décembre [Number days of stock-out of microscopy test in December]**

Question Type= numeric

Data Field Name : Number_stockout_ge_dec

Min 0

Max 31

**71.Durant l’année 2018, le centre de santé a-t-il connu une rupture de stock des tests de diagnostic rapide (TDR) dans un des mois ? [In 2018, the health centre has had a stock-out of Rapid Diagnostic test of malaria (RDT) at least in one month?]**

Question Type= radio

Data Field Name : During_2018_stockout_rdt

Choose one response:

- Oui (Yes)

- Non (No) If this response, jump to 85

**72.Si oui, à quels mois y a-t-il eu rupture de stock des tests de diagnostic rapide (TDR) et pour combien de jours ? (veuillez cocher les mois concernés) [In which month there was a stock-out of Rapid Diagnostic test of malaria (RDT) and how many days? (Please check the corresponding months)]**

Question Type= checkbox

Data Field Name : Which_month_stockout_rdt

Choose all that apply:

- Janvier (January)

- Février (February)

- Mars (March)

- Avril (April)

- Mai (May)

- Juin (June)

- Juillet (July)

- Août (August)

- Septembre (September)

- Octobre (October)

- Novembre (November)

- Décembre (December)

**73.Nombre de jours de rupture de stock TDR janvier [Number days of stock-out of RDT in January]**

Question Type= numeric

Data Field Name : Number_stockout_rdt_jan

Min 0

Max 31

**74.Nombre de jours de rupture de stock TDR février [Number days of stock-out of RDT in February]**

Question Type= numeric

Data Field Name : Number_stockout_rdt_feb

Min 0

Max 28

**75.Nombre de jours de rupture de stock TDR mars [Number days of stock-out of RDT in March]**

Question Type= numeric

Data Field Name : Number_stockout_rdt_march

Min 0

Max 30

**76.Nombre de jours de rupture de stock TDR avril [Number days of stock-out of RDT in April]**

Question Type= numeric

Data Field Name : Number_stockout_rdt_april

Min 0

Max 31

**77.Nombre de jours de rupture de stock TDR mai [Number days of stock-out of RDT in May]**

Question Type= numeric

Data Field Name : Number_stockout_rdt_may

Min 0

Max 30

**78.Nombre de jours de rupture de stock TDR juin [Number days of stock-out of RDT in June]**

Question Type= numeric

Data Field Name : Number_stockout_rdt_june

Min 0

Max 31

**79.Nombre de jours de rupture de stock TDR juillet [Number days of stock-out of RDT in July]**

Question Type= numeric

Data Field Name : Number_stockout_rdt_july

Min 0

Max 30

**80.Nombre de jours de rupture de stock TDR août [Number days of stock-out of RDT in August]**

Question Type= numeric

Data Field Name : Number_stockout_rdt_august

Min 0

Max 31

**81.Nombre de jours de rupture de stock TDR septembre [Number days of stock-out of RDT in September]**

Question Type= numeric

Data Field Name : Number_stockout_rdt_sept

Min 0

Max 30

**82.Nombre de jours de rupture de stock TDR octobre [Number days of stock-out of RDT in October]**

Question Type= numeric

Data Field Name : Number_stockout_rdt_oct

Min 0

Max 31

**83.Nombre de jours de rupture de stock TDR novembre [Number days of stock-out of RDT in November]**

Question Type= numeric

Data Field Name : Number_stockout_rdt_nov

Min 0

Max 30

**84.Nombre de jours de rupture de stock TDR décembre [Number days of stock-out of RDT in December]**

Question Type= numeric

Data Field Name : Number_stockout_rdt_dec

Min 0

Max 31

**85.Le personnel responsable de la prise en charge des cas du paludisme a-t-il reçu une formation sur la pratique de test de diagnostic rapide (TDR) ? [The staff responsible for malaria case management has received a training on the practice of Rapid Diagnostic test (RDT)?]**

Question Type= radio

Data Field Name : Staff_case_managt_trained_rdt

Choose one response:

- Oui (Yes)

- Non (No)

**86.Le personnel responsable de la prise en charge des cas du paludisme a-t-il reçu une formation sur la pratique de test de diagnostic microbiologique (GE) ? [The staff responsible for malaria case management has received a training on the microscopy dignaistic test?]**

Question Type= radio

Data Field Name : Staff_case_managt_traine_micro

Choose one response:

- Oui (Yes)

- Non (No)

**87.Le centre de santé a-t-il le guide ou protocole écrit de prise en charge du paludisme ? [The health centre has a guideline or written protocol on simple malaria case management?]**

Question Type= radio

Data Field Name : Clinic_guidelines_protocol

Choose one response:

- Oui, ils sont affichés au mur du centre de santé selon la recommandation en vigueur (Posted_recommended_practice)

- Oui, mais ils ne sont pas visibles au centre de santé (Yes_not_visible_clinic)

- Non, le centre de santé respecte le protocole mais il n’est pas documenté (Follow_protocol_not_recorded_writing)

- Non (No)

**88.Le personnel responsable de la prise en charge des cas du paludisme a-t-il été formé dans ce domaine ? [The staff responsible for malaria case management has received a training on that thematic (protocol of simple malaria case management)]**

Question Type= radio

Data Field Name : Staff_trained_case_management

Choose one response:

- Oui (Yes)

- Non (No)

**89.Durant l’année 2018, le centre de santé a-t-il reçu pour chaque mois une supervision du MSP/PNLP/district ? [In 2018, the health centre received for each month a supervision visit from MOH/NMCP/district?]**

Question Type= radio

Data Field Name : Clinic_inspect_gov_nmcp_2018

Choose one response:

- Oui (Yes) If this response, jump to 92

- Non (No)

**90.Si non, veuillez indiquer pourquoi [If no, please mention why]**

Question Type= Text

Data Field Name : No_describe_why

**91.Sinon, quels mois la supervision n’a pas eu lieu ? (veuillez cocher les mois concernés) [If no, which months the supervision visit did not happened?]**

Question Type= checkbox

Data Field Name : Month_no_inspection

Choose all that apply:

- Janvier (January)

- Février (February)

- Mars (March)

- Avril (April)

- Mai (May)

- Juin (June)

- Juillet (July)

- Août (August)

- Septembre (September)

- Octobre (October)

- Novembre (November)

- Décembre (December)

**92.Durant l’année 2018, la supervision effectué par le MSP/PNLP/district a-t-elle notifiée des problèmes identifiés ? [In the 2018, the supervision visit of the MOH/NMCP/district noticed a problem?]**

Question Type= radio

Data Field Name : Clinic_inspect_gov_nmcp_pbme

Choose one response:

- Oui (Yes)

- Non (No) If this response, jump to 94

- N/A (ex il n'y a pas eu de visite de supervision en 2018) (N_A_no_inspection) If this response, jump to 94

**93.Si oui, veuillez décrire les problèmes [If yes, please describe the problems]**

Question Type= Text

Data Field Name : Yes_describe_pbme

**94.Durant l’année 2018, il y a-t-il eu de rupture de stock en médicaments antipaludiques dans un des mois ? [In 2018, did a stock-out of antimalaria drug happened at least in one month?]**

Question Type= radio

Data Field Name : During_2018_stockout_antimalar

Choose one response:

- Oui (Yes)

- Non (No) If this response, jump to 108

**95.Si oui, à quels mois y a-t-il eu rupture de stock des traitements antipaludiques et pour combien de jours ? (veuillez cocher les mois concernés) [In which month there was a stock-out of antimalaria drug and how many days? (Please check the corresponding months)]**

Question Type= checkbox

Data Field Name : Which_month_stockout_antimalar

Choose all that apply:

- Janvier (January)

- Février (February)

- Mars (March)

- Avril (April)

- Mai (May)

- Juin (June)

- Juillet (July)

- Août (August)

- Septembre (September)

- Octobre (October)

- Novembre (November)

- Décembre (December)

**96.Nombre de jours de rupture de stock traitements antipaludiques janvier [Number days of stock-out of antimalaria drug in January]**

Question Type= numeric

Data Field Name : Number_stockout_antimalar_jan

Min 0

Max 31

**97.Nombre de jours de rupture de stock traitements antipaludiques février [Number days of stock-out of antimalaria drug in February]**

Question Type= numeric

Data Field Name : Number_stockout_antimalar_feb

Min 0

Max 28

**98.Nombre de jours de rupture de stock traitements antipaludiques mars [Number days of stock-out of antimalaria drug in March]**

Question Type= numeric

Data Field Name : Number_stockout_antimala_march

Min 0

Max 30

**99.Nombre de jours de rupture de stock traitements antipaludiques avril [Number days of stock-out of antimalaria drug in April]**

Question Type= numeric

Data Field Name : Number_stockout_antimala_april

Min 0

Max 31

**100.Nombre de jours de rupture de stock traitements antipaludiques mai [Number days of stock-out of antimalaria drug in May]**

Question Type= numeric

Data Field Name : Number_stockout_antimalar_may

Min 0

Max 30

**101.Nombre de jours de rupture de stock traitements antipaludiques juin [Number days of stock-out of antimalaria drug in June]**

Question Type= numeric

Data Field Name : Number_stockout_antimalar_june

Min 0

Max 31

**102.Nombre de jours de rupture de stock traitements antipaludiques juillet [Number days of stock-out of antimalaria drug in July]**

Question Type= numeric

Data Field Name : Number_stockout_antimalar_july

Min 0

Max 30

**103.Nombre de jours de rupture de stock traitements antipaludiques août [Number days of stock-out of antimalaria drug in August]**

Question Type= numeric

Data Field Name : Number_stockout_antimal_august

Min 0

Max 31

**104.Nombre de jours de rupture de stock traitements antipaludiques septembre [Number days of stock-out of antimalaria drug in September]**

Question Type= numeric

Data Field Name : Number_stockout_antimalar_sept

Min 0

Max 30

**105.Nombre de jours de rupture de stock traitements antipaludiques octobre [Number days of stock-out of antimalaria drug in October]**

Question Type= numeric

Data Field Name : Number_stockout_antimalar_oct

Min 0

Max 31

**106.Nombre de jours de rupture de stock traitements antipaludiques novembre [Number days of stock-out of antimalaria drug in November]**

Question Type= numeric

Data Field Name : Number_stockout_antimalar_nov

Min 0

Max 30

**107.Nombre de jours de rupture de stock traitements antipaludiques décembre [Number days of stock-out of antimalaria drug in December]**

Question Type= numeric

Data Field Name : Number_stockout_antimalar_dec

Min 0

Max 31

**108.Quelles améliorations peuvent-elles être appliquées à la collecte, l’enregistrement ou le rapportage des données du paludisme ? Veuillez décrire [Which improving can be apply to the malaria data collecting, recording or sharing? Please describe]**

Question Type= Text

Data Field Name : Improvement_data_collection

**109.Durant l’année 2018, il y a-t-il eu de rupture de stock de registre dans un des mois ? [In 2018, did a stock-out of register happened at least in one month?]**

Question Type= radio

Data Field Name : During_2018_stockout_register

Choose one response:

- Oui (Yes)

- Non, les registres étaient pleines mais nous avons fait des copies (ex photocopie) (No_register_full_copied_ourselves) If this response, jump to 123

- Non (No) If this response, jump to 123

**110.Si oui, à quels mois y a-t-il eu rupture de stock des registres et pour combien de jours ? (veuillez cocher les mois concernés) [In which month there was a stock-out of register and how many days? (Please check the corresponding months)]**

Question Type= checkbox

Data Field Name : Which_month_stockout_register

Choose all that apply:

- Janvier (January)

- Février (February)

- Mars (March)

- Avril (April)

- Mai (May)

- Juin (June)

- Juillet (July)

- Août (August)

- Septembre (September)

- Octobre (October)

- Novembre (November)

- Décembre (December)

**111.Nombre de jours de rupture de stock des registres janvier [Number days of stock-out of register in January]**

Question Type= numeric

Data Field Name : Number_stockout_register_jan

Min 0

Max 31

**112.Nombre de jours de rupture de stock des registres février [Number days of stock-out of register in February]**

Question Type= numeric

Data Field Name : Number_stockout_register_feb

Min 0

Max 28

**113.Nombre de jours de rupture de stock des registres mars [Number days of stock-out of register in March]**

Question Type= numeric

Data Field Name : Number_stockout_register_march

Min 0

Max 31

**114.Nombre de jours de rupture de stock des registres avril [Number days of stock-out of register in April]**

Question Type= numeric

Data Field Name : Number_stockout_register_april

Min 0

Max 30

**115.Nombre de jours de rupture de stock des registres mai [Number days of stock-out of register in May]**

Question Type= numeric

Data Field Name : Number_stockout_register_may

Min 0

Max 31

**116.Nombre de jours de rupture de stock des registres juin [Number days of stock-out of register in June]**

Question Type= numeric

Data Field Name : Number_stockout_register_june

Min 0

Max 30

**117.Nombre de jours de rupture de stock des registres juillet [Number days of stock-out of register in July]**

Question Type= numeric

Data Field Name : Number_stockout_register_july

Min 0

Max 31

**118.Nombre de jours de rupture de stock des registres août [Number days of stock-out of register in August]**

Question Type= numeric

Data Field Name : Number_stockout_register_augus

Min 0

Max 31

**119.Nombre de jours de rupture de stock des registres septembre [Number days of stock-out of register in September]**

Question Type= numeric

Data Field Name : Number_stockout_register_sept

Min 0

Max 30

**120.Nombre de jours de rupture de stock des registres octobre [Number days of stock-out of register in October]**

Question Type= numeric

Data Field Name : Number_stockout_register_oct

Min 0

Max 31

**121.Nombre de jours de rupture de stock des registres novembre [Number days of stock-out of register in November]**

Question Type= numeric

Data Field Name : Number_stockout_register_nov

Min 0

Max 30

**122.Nombre de jours de rupture de stock des registres décembre [Number days of stock-out of register in December]**

Question Type= numeric

Data Field Name : Number_stockout_register_dec

Min 0

Max 31

**123.Durant l’année 2018, il y a-t-il eu de rupture de stock de formulaire RMA dans un des mois ? [In 2018, did a stock-out of monthly report of activities form happened at least in one month?]**

Question Type= radio

Data Field Name : During_2018_stockout_HMIS_form

Choose one response:

- Oui (Yes)

- Non, les formulaires étaient pleines mais nous avons fait des copies (ex photocopie) (No_form_full_copied_ourselves) If this response, jump to 137

- Non (No) If this response, jump to 137

**124.Si oui, à quels mois y a-t-il eu rupture de stock des formulaires RMA et pour combien de jours ? (veuillez cocher les mois concernés) [In which month there was a stock-out of monthly report of activities form and how many days? (Please check the corresponding months)]**

Question Type= checkbox

Data Field Name : Which_month_stockout_form

Choose all that apply:

- Janvier (January)

- Février (February)

- Mars (March)

- Avril (April)

- Mai (May)

- Juin (June)

- Juillet (July)

- Août (August)

- Septembre (September)

- Octobre (October)

- Novembre (November)

- Décembre (December)

**125.Nombre de jours de rupture de stock des formulaires janvier [Number days of stock-out of monthly report of activities form in January]**

Question Type= numeric

Data Field Name : Number_stockout_form_jan

Min 0

Max 30

**126.Nombre de jours de rupture de stock des formulaires février [Number days of stock-out of monthly report of activities form in February]**

Question Type= numeric

Data Field Name : Number_stockout_form_feb

Min 0

Max 28

**127.Nombre de jours de rupture de stock des formulaires mars [Number days of stock-out of monthly report of activities form in March]**

Question Type= numeric

Data Field Name : Number_stockout_form_march

Min 0

Max 31

**128.Nombre de jours de rupture de stock des formulaires avril [Number days of stock-out of monthly report of activities form in April]**

Question Type= numeric

Data Field Name : Number_stockout_form_april

Min 0

Max 30

**129.Nombre de jours de rupture de stock des formulaires mai [Number days of stock-out of monthly report of activities form in May]**

Question Type= numeric

Data Field Name : Number_stockout_form_may

Min 0

Max 31

**130.Nombre de jours de rupture de stock des formulaires juin [Number days of stock-out of monthly report of activities form in June]**

Question Type= numeric

Data Field Name : Number_stockout_form_june

Min 0

Max 30

**131.Nombre de jours de rupture de stock des formulaires juillet [Number days of stock-out of monthly report of activities form in July]**

Question Type= numeric

Data Field Name : Number_stockout_form_july

Min 0

Max 31

**132.Nombre de jours de rupture de stock des formulaires août [Number days of stock-out of monthly report of activities form in August]**

Question Type= numeric

Data Field Name : Number_stockout_form_august

Min 0

Max 31

**133.Nombre de jours de rupture de stock des formulaires septembre [Number days of stock-out of monthly report of activities form in September]**

Question Type= numeric

Data Field Name : Number_stockout_form_sept

Min 0

Max 30

**134.Nombre de jours de rupture de stock des formulaires octobre [Number days of stock-out of monthly report of activities form in October]**

Question Type= numeric

Data Field Name : Number_stockout_form_oct

Min 0

Max 30

**135.Nombre de jours de rupture de stock des formulaires novembre [Number days of stock-out of monthly report of activities form in November]**

Question Type= numeric

Data Field Name : Number_stockout_form_nov

Min 0

Max 30

**136.Nombre de jours de rupture de stock des formulaires décembre [Number days of stock-out of monthly report of activities form in December]**

Question Type= numeric

Data Field Name : Number_stockout_form_dec

Min 0

Max 31

**137.Durant l’année 2018, il y a-t-il eu de rupture de stock de formulaire RMP dans un des mois ? [In 2018, did a stock-out of monthly report of malaria form happened at least in one month?]**

Question Type= radio

Data Field Name : During_2018_stockout_RMP

Choose one response:

- Oui (Yes)

- Non, les formulaires étaient pleines mais nous avons fait des copies (ex photocopie) (No_form_full_copied_ourselves) If this response, jump to 151

- Non (No) If this response, jump to 151

**138.Si oui, à quels mois y a-t-il eu rupture de stock des formulaires RMP et pour combien de jours ? (veuillez cocher les mois concernés) [In which month there was a stock-out of monthly report of malaria form and how many days? (Please check the corresponding months)]**

Question Type= checkbox

Data Field Name : Which_month_stockout_rmp

Choose all that apply:

- Janvier (January)

- Février (February)

- Mars (March)

- Avril (April)

- Mai (May)

- Juin (June)

- Juillet (July)

- Août (August)

- Septembre (September)

- Octobre (October)

- Novembre (November)

- Décembre (December)

**139.Nombre de jours de rupture de stock des rmp janvier [Number days of stock-out of monthly report of malaria form in January]**

Question Type= numeric

Data Field Name : Number_stockout_rmp_jan

Min 0

Max 31

**140.Nombre de jours de rupture de stock des rmp février [Number days of stock-out of monthly report of malaria form in February]**

Question Type= numeric

Data Field Name : Number_stockout_rmp_feb

Min 0

Max 28

**141.Nombre de jours de rupture de stock des rmp mars [Number days of stock-out of monthly report of malaria form in March]**

Question Type= numeric

Data Field Name : Number_stockout_rmp_march

Min 0

Max 31

**142.Nombre de jours de rupture de stock des rmp avril [Number days of stock-out of monthly report of malaria form in April]**

Question Type= numeric

Data Field Name : Number_stockout_rmp_april

Min 0

Max 30

**143.Nombre de jours de rupture de stock des rmp mai [Number days of stock-out of monthly report of malaria form in May]**

Question Type= numeric

Data Field Name : Number_stockout_rmp_may

Min 0

Max 31

**144.Nombre de jours de rupture de stock des rmp juin [Number days of stock-out of monthly report of malaria form in June]**

Question Type= numeric

Data Field Name : Number_stockout_rmp_june

Min 0

Max 30

**145.Nombre de jours de rupture de stock des rmp juillet [Number days of stock-out of monthly report of malaria form in July]**

Question Type= numeric

Data Field Name : Number_stockout_rmp_july

Min 0

Max 31

**146.Nombre de jours de rupture de stock des rmp août [Number days of stock-out of monthly report of malaria form in August]**

Question Type= numeric

Data Field Name : Number_stockout_rmp_aug

Min 0

Max 31

**147.Nombre de jours de rupture de stock des rmp septembre [Number days of stock-out of monthly report of malaria form in September]**

Question Type= numeric

Data Field Name : Number_stockout_rmp_sept

Min 0

Max 30

**148.Nombre de jours de rupture de stock des rmp octobre [Number days of stock-out of monthly report of malaria form in October]**

Question Type= numeric

Data Field Name : Number_stockout_rmp_oct

Min 0

Max 31

**149.Nombre de jours de rupture de stock des rmp novembre [Number days of stock-out of monthly report of malaria form in November]**

Question Type= numeric

Data Field Name : Number_stockout_rmp_nov

Min 0

Max 30

**150.Nombre de jours de rupture de stock des rmp décembre [Number days of stock-out of monthly report of malaria form in December]**

Question Type= numeric

Data Field Name : Number_stockout_rmp_dec

Min 0

Max 31

**151.Durant l’année 2018, pour tous les mois avez-vous reçu des motivations financières pour le rapportage du rapport mensuel du paludisme (RMP) ? [In 2018, for each month did you received incentive related to the montly report on malaria data?]**

Question Type= radio

Data Field Name : During_2018_incentive_rmp

Choose one response:

- Oui (Yes) If this response, jump to 165

- Non (No)

**152.Sinon, à quels mois avez-vous eu des arriérés et pour combien de jours ? (veuillez cocher les mois concernés) [If no, in which months are there arrears of incentive payment and how long? (Please check the corresponding months)]**

Question Type= checkbox

Data Field Name : Which_month_arrears_incentive

Choose all that apply:

- Janvier (January)

- Février (February)

- Mars (March)

- Avril (April)

- Mai (May)

- Juin (June)

- Juillet (July)

- Août (August)

- Septembre (September)

- Octobre (October)

- Novembre (November)

- Décembre (December)

**153.Nombre de jours d'arriérés janvier [Number days of arrears in January]**

Question Type= numeric

Data Field Name : Number_day_arrears_jan

Min 0

Max 31

**154.Nombre de jours d'arriérés février [Number days of arrears in February]**

Question Type= numeric

Data Field Name : Number_day_arrears_feb

Min 0

Max 28

**155.Nombre de jours d'arriérés mars [Number days of arrears in March]**

Question Type= numeric

Data Field Name : Number_arrears_march

Min 0

Max 31

**156.Nombre de jours d'arriérés avril [Number days of arrears in April]**

Question Type= numeric

Data Field Name : Number_day_arrears_april

Min 0

Max 30

**157.Nombre de jours d'arriérés mai [Number days of arrears in May]**

Question Type= numeric

Data Field Name : Number_day_arrears_may

Min 0

Max 31

**158.Nombre de jours d'arriérés juin [Number days of arrears in June]**

Question Type= numeric

Data Field Name : Number_day_arrears_june

Min 0

Max 30

**159.Nombre de jours d'arriérés juillet [Number days of arrears in July]**

Question Type= numeric

Data Field Name : Number_day_arrears_july

Min 0

Max 31

**160.Nombre de jours d'arriérés août [Number days of arrears in August]**

Question Type= numeric

Data Field Name : Number_day_arrears_august

Min 0

Max 31

**161.Nombre de jours d'arriérés septembre [Number days of arrears in September]**

Question Type= numeric

Data Field Name : Number_day_arrears_sept

Min 0

Max 30

**162.Nombre de jours d'arriérés octobre [Number days of arrears in October]**

Question Type= numeric

Data Field Name : Number_day_arrears_oct

Min 0

Max 31

**163.Nombre de jours d'arriérés novembre [Number days of arrears in November]**

Question Type= numeric

Data Field Name : Number_day_arrears_nov

Min 0

Max 30

**164.Nombre de jours d'arriérés décembre [Number days of arrears in December]**

Question Type= numeric

Data Field Name : Number_day_arrears_dec

Min 0

Max 31

**165. Fin du questionnaire: veuillez remercier l'interviewé(e) et passer au centre de santé suivant [End of questionnaire : please thank the interviewee and go to next health centre]**
